# Design of Detection Training Equipment for Penetrating Radiation Field from Nuclear Fuel in a Tunnel Environment

**DOI:** 10.3390/s26041194

**Published:** 2026-02-12

**Authors:** Gui Huang, Haiyan Li, Biao Li, Fei Wu, Ming Guo, Xin Xie

**Affiliations:** Naval University of Engineering, Wuhan 430033, China

**Keywords:** neutron/gamma radiation field, radiation detection, simulation training, inertial sensor

## Abstract

To address the problems existing in nuclear reactor accident emergency training, a design scheme and system prototype of radiation detection training equipment for penetrating radiation fields in enclosed spaces, based on inertial sensors and wireless Bluetooth communication is proposed. First, the penetrating radiation field is modeled. On this basis, a calculation model of the neutron/*γ* dose equivalent rate is established. This model is based on the motion path of simulated radiation detection equipment. Second, the MPU6050 inertial sensor is designed and developed. It monitors the three-axis acceleration and three-axis angular acceleration values in real time. This enables the indoor positioning function of the simulated detection training equipment. The Digital Motion Processor (DMP) filtering algorithm is used to process the measured data. This improves the detection accuracy. Finally, a Bluetooth communication module is designed and developed. It transmits the detected position data to the main control computer in real time. The main control computer performs calculation and analysis to obtain the radiation intensity value. This value is sent to the Arduino controller. The Arduino controller controls the display of the value on the liquid crystal screen. Experimental verification is carried out. Experimental verification indicates that the maximum error of the system’s three-dimensional spatial positioning is 0.08 m, the mean relative error of the radiation dose rate simulation is 4.81%, and the maximum relative error is 7.8%. The system relatively accurately achieves radiation dose simulation and radiation source localization according to different working modes, providing a high cost-effectiveness training method for radiation detection training with high safety and good economy.

## 1. Introduction

Since the 2011 Fukushima nuclear power plant accident in Japan, nuclear radiation safety has remained a global focus [1,2,3,4]. The accident brought problems such as nuclear radiation pollution control and public health. These problems keep nuclear radiation safety a key part of global security issues. Nuclear radiation safety control is a challenge for the community with a shared future for mankind. Professional emergency rescue plays a crucial role in addressing the challenges of nuclear accidents. Nuclear fuel radiation reconnaissance and detection training is a very important link [5,6,7].

Many practical problems exist in conventional nuclear radiation detection training. For example, considering the personal safety of trainers, radioactive sources with high radiation dose rates cannot be used as training sources. These problems greatly affect training effectiveness. Therefore, using simulation methods to conduct radiation detection training has become one of the main means for training nuclear professionals. Tlaczala [8], Tiftikci [9], and James [10] constructed radiation detection training systems or laboratories through virtual reality or physical simulation technologies. Danilo [11] achieved precise positioning of equipment through a hybrid method of artificial intelligence and finite elements. This provides a reference for improving the accuracy of hybrid radiation field modeling methods. Zhang [12] and Guo [13] constructed nuclear emergency response and rescue training systems using virtual reality technology. These systems enable simulated radiation detection training during nuclear emergencies. Jiang [14] combined global positioning technology with computers. This combination enables the simulation of ground radiation fields after nuclear explosions. It solves the problem of radiation reconnaissance. Lin [15] adopted a passive method to enable numerical simulation, detection and analysis of nuclear material radiation fields. Zhang [16] developed a radiation monitoring system training simulator using a hardware-in-the-loop simulation technology route. All the above simulated detection equipment has achieved good application results. Among them, training equipment developed using physical simulation schemes is mainly used for outdoor radiation detection training. Outdoor areas are wide with large target ranges. The requirements for measurement error accuracy are not high. Additionally, there is no need to consider the impact of indoor signal shielding. However, for physical simulation training of radiation detection in tunnel environments, the limited indoor space and the impact of signal shielding make it difficult to guarantee the measurement accuracy and real-time performance of existing technical schemes. Existing indoor positioning technologies include Bluetooth Low Energy (BLE) [17], ultrawideband (UWB) [18], pedestrian dead reckoning (PDR) [19], Quick Response (QR) [20], and radio frequency identification (RFID) [21]. Most of these existing technologies have the advantage of high prediction accuracy. However, these technologies still have some shortcomings. The coverage of BLE technology has certain limitations. UWB technology has high costs and cannot meet existing needs easily. PDR technology has large cumulative errors. Its positioning accuracy decreases with time. QR technology cannot meet the needs of nuclear radiation detection training. RFID technology is greatly affected by environmental interference factors. In recent years, researchers have proposed hybrid systems that combine inertial measurement units with wireless communication. Zhang [22] integrated an IMU with a Hall sensor array. This integration achieves high-precision positioning. However, the effective positioning distance is limited. Vedaei [23] integrated an IMU with sidewall cameras. This integration can accurately capture 3D trajectories without external reference equipment. However, multiple modules consume high power and have poor battery life. Wu [24] integrated an IMU with RSS-based OWP. This integration reduces positioning errors. However, data synchronization poses challenges. Although existing indoor positioning technologies are relatively mature, many practical problems exist in nuclear radiation detection training.

Existing single technical schemes (BLE, UWB, PDR, QR, RFID) cannot simultaneously meet the requirements of anti-shielding, low cost, and low cumulative error in enclosed spaces. Hybrid technical schemes have shortcomings such as short detection distance, poor battery life, and low practicality. These shortcomings greatly affect the detection performance of simulated detection of nuclear fuel penetrating radiation fields in enclosed spaces. To address this, this paper designs a scheme for simulated radiation detection training equipment in indoor positions based on inertial sensors and wireless Bluetooth communication. It simulates the radiation dose rate values formed by radiation sources in space through changes in spatial distance. This effectively simulates the radiation field detection training process.

## 2. Methods

The key to conducting radiation detection training in an enclosed space environment lies in radiation field simulation and radiation source localization. Therefore, this paper focuses on the design of a radiation field simulation and detection system, with the core aim of addressing these two key technical issues.

### 2.1. Modeling of Indoor Radiation Field Intensity

**Figure 1:**
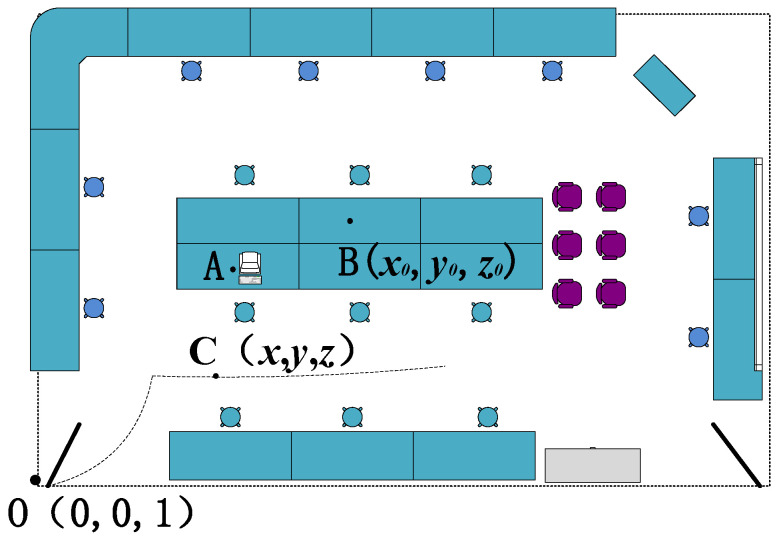
Schematic diagram of the laboratory.

The intensity of the indoor radiation field is determined by the characteristics of the radiation source and environmental factors. In this paper, the simulated enclosed space is set as a specific simulated laboratory, and the layout of the laboratory is shown in Figure 1. Point A is the location where the main control computer of the radiation simulation detection equipment is situated.

This study establishes a laboratory spatial coordinate system *O_xyz_*, with the origin *O* at (0, 0, 1), the *OZ* axis pointing vertically upward, the *OX* axis pointing horizontally to the right, and the *OY* axis pointing forward; at the initial time, the radiation simulation and detection equipment initializes at *O*(0, 0, 1), and the simulation assumes a point source, where the host computer sets the source position at *B*(*x*_0_, *y*_0_, *z*_0_), as shown in Figure 1; as the radiation detection equipment moves along the dashed path in Figure 1, the radiation intensity at any position *C*(*x*, *y*, *z*) depends on the spatial distance *R* between *B* and *C*, the medium type, and the medium distribution; to improve simulation accuracy, the *γ* and neutron radiation fields use the Monte Carlo particle transport code SuperMC, which simulates the photon transport process and calculates the neutron radiation field, the gamma radiation field, and the mixed gamma–neutron radiation field, with the primary metric being the distribution of the neutron/*γ* dose equivalent rate in the laboratory space under the assumed source conditions; the simulation workflow includes generating the geometric model via CAD modeling, producing the physical model via parametric physical modeling, performing three-dimensional radiation field simulation to obtain a virtual radiation environment, and referencing [25,26].

### 2.2. Principle of Calculation of the Motion Path of Radiation Simulation Detection Equipment

**Figure 2:**
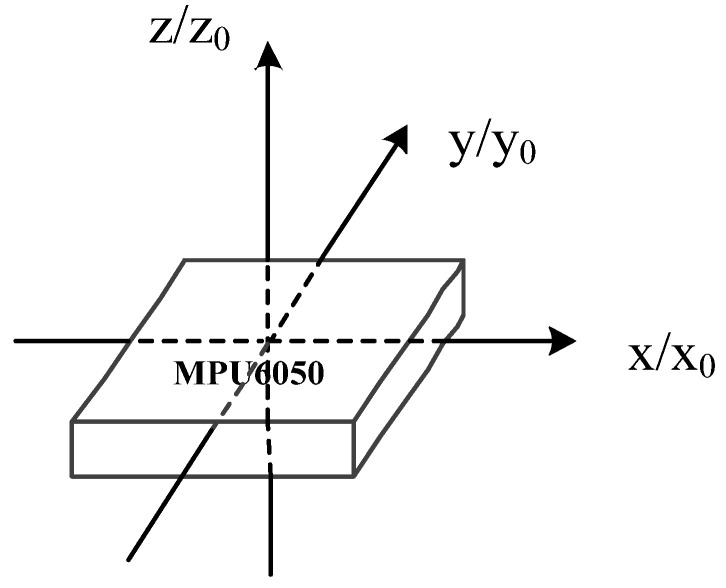
Schematic diagram of the inertial coordinate system and the MPU6050 coordinate system.

MPU6050 inertial sensors are widely used, as shown in Figure 2. The sensor offers a small size, low cost, and low power consumption. It also provides attitude and motion data without additional multi-sensor suites, which simplifies system integration. Although the sensor has drawbacks such as zero-offset, these can be suppressed by optimized cooperative strategies. It aligns closely with the application scenarios and core requirements of enclosed-space nuclear radiation detection training equipment and fully meets the accuracy requirements for radiation detection training. Under ideal conditions, it is assumed that the MPU6050 inertial sensor remains horizontally level, with its *y*-axis always pointing forward, the *x*-axis horizontal to the right (the right-hand direction when moving forward), and the *z*-axis vertical upward. In this case, the inertial coordinate system *x*_0_*y*_0_*z*_0_ coincides with the MPU6050 coordinate system *xyz*. The system computes the spatial trajectory and direction angle in real time from the three-axis acceleration values [27,28].

**Figure 3:**
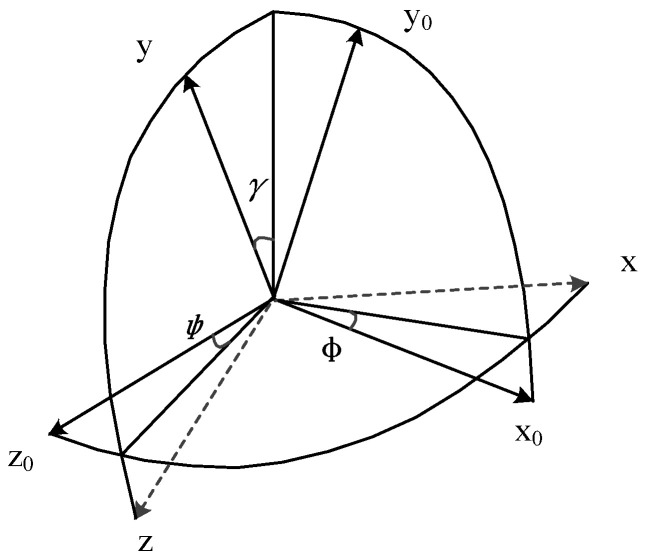
Schematic diagram of coordinate conversion.

The above two coordinate systems are not completely coincident in actual motion, as shown in Figure 3. In the figure, γ, φ, ψ respectively represent the rotation angle around the three axes of *X*, *Y*, and *Z* (the meaning of the angle is determined by the rotation order), which are called pitch, yaw, and roll angles.

To transform the MPU6050 coordinate system into the inertial coordinate system of the simulated detection device, this paper adopts a rotation sequence: first around the *Z*_0_-axis, second around the transitional *Y*_0_′-axis, and third around the transitional *X*′-axis. This means rotating initially around the positive *z*-axis by an angle *φ*, then rotating around the positive transitional *Y*_0_′-axis by an angle *φ*, and finally rotating around the transitional *X*′-axis by an angle *γ*. The ZYX rotation sequence matches the pattern of human motion attitude changes. First, the yaw angle (rotation around the *Z*_0_-axis) determines the travel direction. Then, the pitch angle (rotation around the transitional *Y*_0_′-axis) and roll angle (rotation around the transitional *X*′-axis) correct for attitude deviations caused by device tilting. This aligns with practical operational logic. Based on this transformation sequence, the directional cosine relationship between the two coordinate systems can be expressed as follows:

(1) $$\left[\begin{array}{c} x\\ y\\ z \end{array}\right]=A_{T}\left[\begin{array}{c} x_{0}\\ y_{0}\\ z_{0} \end{array}\right]$$

where

(2) $$\;A_{T}=C_{T3}C_{T2}C_{T1}$$

The three rotation matrices are:

(3) $$C_{T1}=\left[\begin{array}{ccc} cos\varphi & sin\varphi & 0\\ -sin\varphi & cos\varphi & 0\\ 0 & 0 & 1 \end{array}\right]$$

(4) $$C_{T2}=\left[\begin{array}{ccc} 1 & 0 & 0\\ 0 & cos\gamma & sin\gamma \\ 0 & -sin\gamma & cos\gamma \end{array}\right]$$

(5) $$C_{T3}=\left[\begin{array}{ccc} cos\psi & 0 & -sin\psi \\ 0 & 1 & 0\\ sin\psi & 0 & cos\psi \end{array}\right]$$

Thus, the rotation matrix for the transformation from the coordinate *O_xyz_* to the coordinate *O_x_*_0*y*0*z*0_ is obtained:

(6) $$AT=\left[\begin{array}{ccc} cos\varphi cos\psi & sin\varphi cos\psi & -sin\psi \\ cos\varphi sin\psi sin\gamma & sin\varphi sin\psi sin\gamma +cos\varphi cos\gamma & cos\psi sin\gamma \\ cos\varphi sin\psi cos\gamma & sin\varphi sin\psi cos\gamma -cos\varphi sin\gamma & cos\psi cos\gamma \end{array}\right]$$

**Figure 4:**
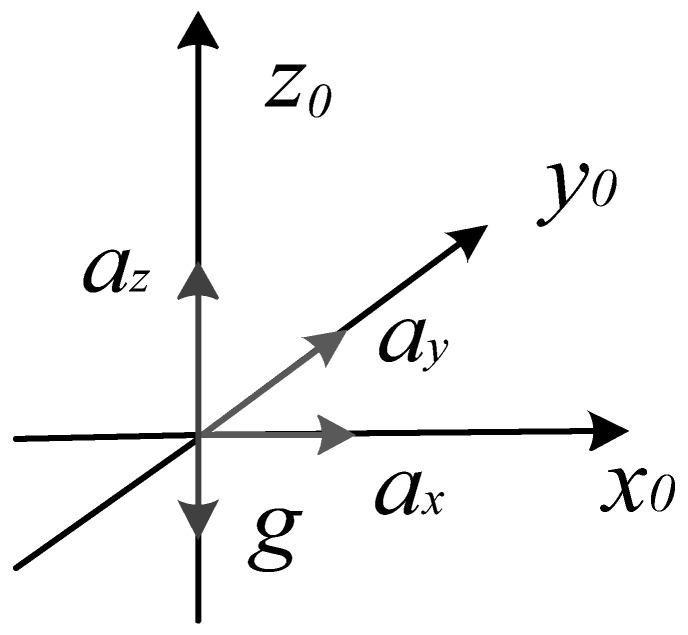
Schematic diagram of acceleration.

Through coordinate transformation, the three-axis linear acceleration in the inertial coordinate system is obtained, which are recorded as *a_x_*, *a_y_*, and *a_z_*. The schematic diagram of the acceleration of the simulated detection device during the movement is shown in Figure 4.

The velocities *v_x_*, *v_y_*, and *v_z_*, and displacements *S_x_*, *S_y_*, and *S_z_* in the three axis directions can be obtained from the knowledge of physics [29,30]. Their calculation formulas are as follows:

(7) $$\left\{\begin{array}{c} v_{x}=v_{0}+\int a_{x}dt\\ v_{y}=v_{0}+\int a_{y}dt\\ v_{z}=v_{0}+\int \left(a_{z}-g\right)dt \end{array}\right.$$

(8) $$\left\{\begin{array}{c} S_{x}=S_{0x}+\int v_{x}dt\\ S_{y}=S_{0y}+\int v_{y}dt\\ S_{z}=S_{0z}+\int v_{z}dt \end{array}\right.$$

where *v*_0_ and *S*_0_ are the initial velocity and initial displacement of the simulated detection device. In this experimental scheme, the simulated detection equipment starts to move from the initial position *S*_0_(0,0,1) at rest, so the above two equations can be simplified as:

(9) $$\left\{\begin{array}{c} v_{x}=\int a_{x}dt\\ v_{y}=\int a_{y}dt\\ v_{z}=\int \left(a_{z}-g\right)dt \end{array}\right.$$

(10) $$\left\{\begin{array}{c} S_{x}=\int v_{x}dt\\ S_{y}=\int v_{y}dt\\ S_{z}=1+\int v_{z}dt \end{array}\right.$$

Finally, the real-time spatial coordinates of the simulated detection equipment are (*S_x_*, *S_y_*, *S_z_*). The distance between the simulated detection equipment and the simulated radiation source (coordinates (*x*_0_, *y*_0_, *z*_0_)) can be calculated by the distance formula between two points.

(11) $$R=\sqrt[{2}]{{\left(x_{0}-S_{x}\right)}^{2}+{\left(y_{0}-S_{y}\right)}^{2}+{\left(z_{0}-S_{z}\right)}^{2}}$$

## 3. Results

### 3.1. Design of Detection Training Equipment

This paper adopts the Arduino UNO R3 produced by Arduino S.r.l. in Italy as the main control chip of the analog detection equipment. It employs the MPU6050 inertial sensor for processing inertial position information and data. An HC-05 Bluetooth module enables communication between the host computer and the radiation simulation detection device. The module’s antenna is externalized and mounted at a 30° tilt relative to the device casing to reduce signal shielding from metal components. Furthermore, an independent shield protects the module, mitigating electromagnetic interference from other electronic devices in the nuclear radiation training environment. The LCD12864 liquid crystal display module updates and displays the radiation dose rate value of the simulated detection equipment in real time. Among them, the MPU6050 sensor adopts the Kalman and DMP motion processor for data output and fusion calculation processing to eliminate errors and compensate for data such as acceleration, angular velocity, three-axis rotational angular velocity, and spatial three-axis displacement. Through the theory of space inertial coordinate and target carrier coordinate transformation, the angular velocity after error elimination and compensation is converted to the corresponding coordinate axes of the moving simulated detection equipment. The three-axis spatial displacement is obtained through integration and compensation, so as to obtain the spatial position information of the simulated detection equipment. Finally, the radiation dose rate value is calculated through the radiation dose rate calculation formula, and the data are displayed on the LCD screen in real time with updates per second.

The MPU6050 employs a static calibration method, with the sensor fixed to a high-precision fixture in a vibration-free, constant-temperature laboratory environment at 25 ± 2 °C to avoid the effects of gravitational interference and temperature drift. Data are acquired at a sampling frequency of 100 Hz. The zero-bias values are written into the sensor registers and subtracted in real time during data acquisition to eliminate fixed offsets. Dynamic uniform linear motion is performed to define the motion trajectory, and the corresponding calibration is carried out. After calibration, the accelerometer zero-bias error is ≤0.05 m/s^2^, and the gyroscope zero-bias stability is ≤0.5°/s. The sensor sampling frequency is set to 100 Hz, the DMP fusion output frequency is 100 Hz, and the DMP is configured with a 42 Hz low-pass filter. The Kalman filter parameters are set to Q = 0.001 and R = 0.1. The Bluetooth transmission rate is 10 Hz, and the screen refresh period is 1 Hz, meeting the requirements for real-time training.

**Figure 5:**
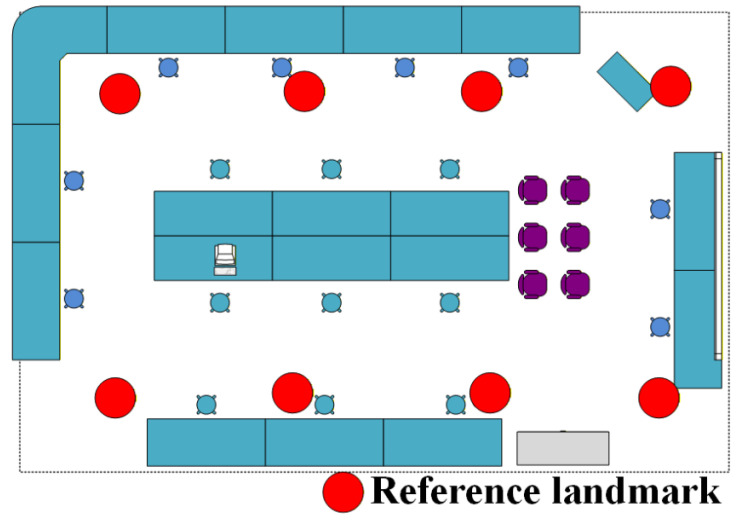
Reference landmark distribution.

During the movement of the positioning module, drift may occur. To avoid the accumulation of errors during training, this study adopts landmark calibration to eliminate such cumulative errors. Specifically, eight reference landmarks are arranged in the laboratory space, and their exact positions are illustrated in Figure 5. Precise three-dimensional coordinates of these reference landmarks are pre-measured. During training, the positioning module is placed at the nearest reference landmark for calibration approximately every 5 min.

The overall design target is an average absolute error in three-dimensional space positioning of ≤0.06 m and a maximum error of ≤0.08 m. For repeated measurements at the same calibration point, the standard deviation of the positioning error is ≤0.01 m, ensuring consistency and reliability of the training data. The relative error between the simulated *γ* dose equivalent rate and the calibrated true value has a mean ≤ 8% and a maximum ≤ 8%, which is below the ±20% allowable deviation specified in the GBZ 128-2002 standard. The system features strong endurance, accurate positioning, and high efficiency.

### 3.2. Overall Design

**Figure 6:**
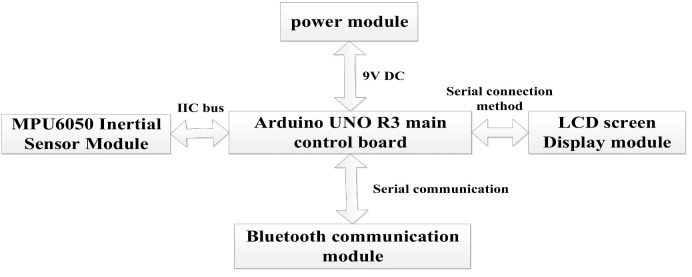
Overall design block diagram of the analog detection equipment.

The overall design block diagram of the analog detection equipment in this work is shown in Figure 6.

The equipment is divided into five parts, namely the Arduino main control board module, MPU6050 inertial module, LCD screen display module, Bluetooth communication module, and power supply module. The Arduino main control module adopts the UNO R3 main control board [31], which is equipped with a power supply filter, a voltage regulator chip, and related circuits. The functions of the main control chip and power module can be achieved simply by powering the entire main control board. A 9V DC power input produced by Delipu Company, located in Guangzhou, Guangdong Province, China, is adopted. Therefore, this section focuses on designing the program flows of the MPU6050 inertial sensor module, Bluetooth communication module, and LCD screen display module [32]. The overall parameters of the device are listed in Table 1.

### 3.3. Each Module Design

#### 3.3.1. MPU6050 Positioning Module Design

**Figure 7:**
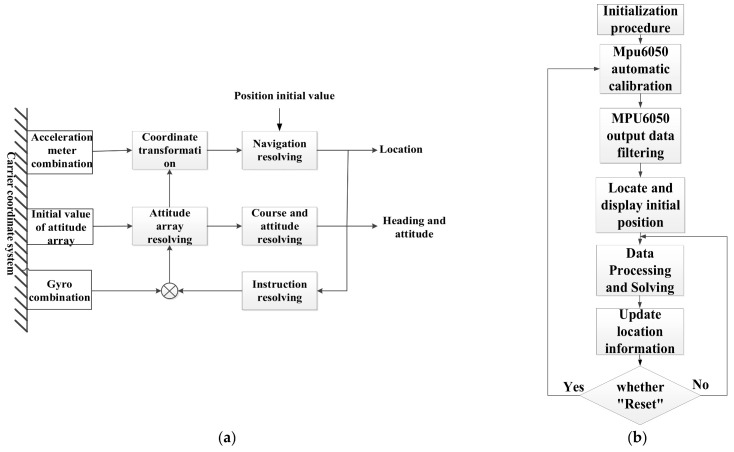
Inertial navigation system. (**a**) Schematic diagram of the inertial navigation system. (**b**) Inertial navigation positioning program flowchart.

In this paper, the indoor positioning scheme is designed as follows: after performing DMP filtering on the data from the MPU6050 three-axis accelerometer and gyroscope, the three-axis rotation angles (i.e., Euler angles) are calculated. The angle values obtained from the two different calculation formulas are fused via a Kalman filter to determine the optimal angle values [33,34]. Then, the rotation vector four-sample algorithm is used to solve the current quaternion, and the rotation matrix is finally derived from the quaternion. Through this matrix, the three-axis acceleration values are converted to the three axes of the moving simulated detection equipment, and the three-dimensional displacements are ultimately obtained through integration with error correction to determine the target position coordinates [35]. Its principle diagram is shown in Figure 7a, and the program flowchart is shown in Figure 7b.

**Figure 8:**
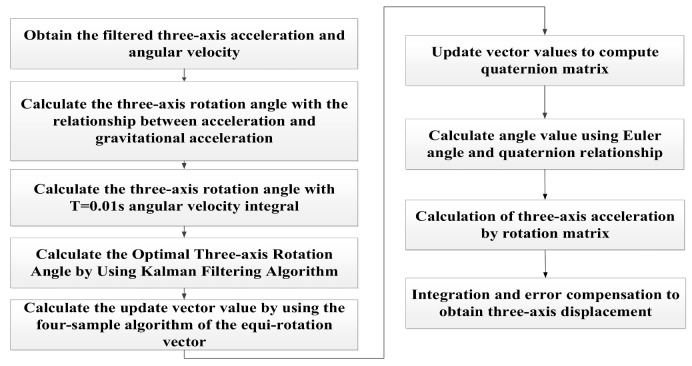
Data processing and calculation flowchart.

The flow of data processing and calculation is shown in Figure 8.

#### 3.3.2. Bluetooth Communication Module Design

In this article, the master-slave connection mode of the HC-05 Bluetooth module is used. The main control machine is in active mode, and it automatically searches for Bluetooth matching and pairing when it is turned on. The analog detection device is in slave mode, waiting to be connected after powering on.

**Figure 9:**
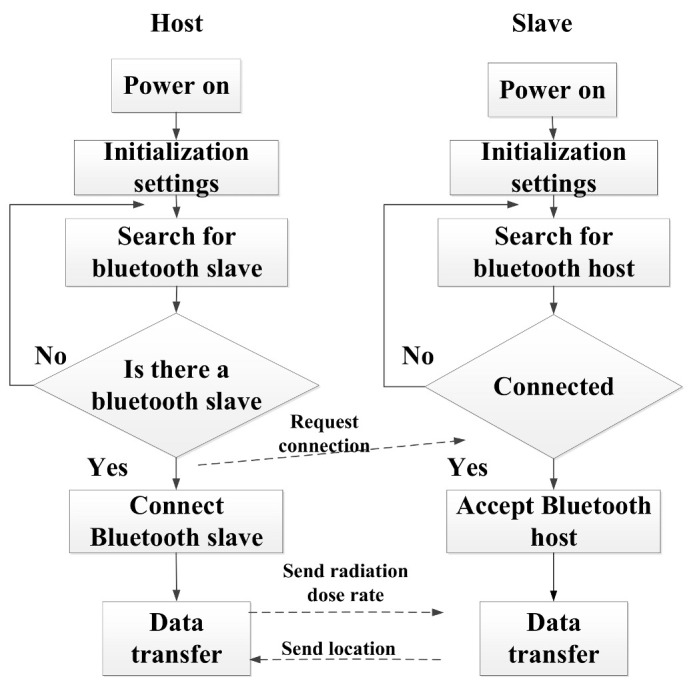
Master-slave Bluetooth communication process.

The main process is: after the master computer and the analog detection device are turned on, the master-slave Bluetooth module starts self-checking. Then, the master module starts to search for a pairable Bluetooth module nearby, and the slave module enters a state of waiting to be connected. When the host module searches for the slave module, it starts to send a request-to-connect command. After the slave receives the command, it starts to connect with the master module. After connection, the Arduino UNO R3 analog detection device main control board sends the processed location information from the slave module to the Bluetooth master module. Next, the Bluetooth master module receives the location information and uploads it to the control terminal of the master computer. After the master computer recognizes and processes the location information, it calls the database and transmits the radiation dose rate value at the location of the simulated detection device to the Bluetooth master. The Bluetooth master sends it to the Bluetooth slave, and the slave receives the data and uploads it to the Arduino UNO R3 master. Eventually, the host computer control screen displays these data. The master-slave Bluetooth communication process is shown in Figure 9.

#### 3.3.3. Screen Display Module Design

After the LCD is powered on, the initialization process is started. The process is as follows: after 40 ms of power-on, the reset port level signal changes from low level to high level, and the reset is started. The first step is to set the function setting and perform the basic command operation of 8-bit data transfer (the minimum operation time of this process should last 100 µs). The second step is to set the display switch control settings. The display mode is set as follows: when *D* = 1, the display mode is the overall display; when *C* = 1, the cursor function is turned on; and when *B* = 1, the cursor position is set to white (the minimum operation time of this process should last 100 µs). The third step is to set the clear screen setting and perform the setting to clear all the contents displayed on the screen. During this process, the D2RAM address is filled, and it starts from 00H again (the minimum operation time of this process should last for 10 ms). The fourth step is to set the input mode setting and perform cursor and shift settings. After the above four steps are set, the initialization process is completed.

After the initialization is completed, the LCD screen will enter the standby mode. In this mode, the screen waits for interruption by other expansion commands. Any expansion command can terminate the standby mode. In this article, when the Arduino UNO R3 main control board receives the radiation dose rate value, it first sends the serial-port read-address command to the LCD. After the LCD control chip receives the address command, it enters the display state from the standby state. Then the main control board sends the display-character internal code to the LCD control chip. After processing, the Chinese characters and radiation dose rate values are displayed at the designated position.

### 3.4. Verifying Equipment Accuracy

Prior to the initiation of training, precomputation is performed using SuperMC, and the results are stored in a data table. In the near-field region, the spatial resolution of the radiation field distribution is 0.05 m, whereas in the far-field region it is 0.1 m. When a measurement point does not coincide with any computation point, the radiation dose rate at the measurement point is calculated using the weighted average of eight adjacent points.

In reactor nuclear accident emergency training, the system primarily trains personnel to locate radioactive sources by detecting penetrating radiation fields in enclosed spaces and to respond promptly. The key performance metrics of interest are positioning accuracy and the credibility of the radiation field simulation.

#### 3.4.1. Spatial Positioning Verification

The system is applied to training for locating radioactive sources and responding to accidents. Considering the movement range of personnel during detection and response operations, a three-dimensional absolute positioning error of no more than 0.2 m does not affect operations or training. Therefore, the permissible positioning error is defined as 0.2 m. To verify the system’s spatial positioning error, this study selected 10 calibration points within the training area, recorded their true coordinates, and had trainees sequentially pass through each point while holding the positioning module to read the measured coordinates. These were compared with the true coordinates to analyze the positioning error (Table 2).

Comparing the true coordinates of the calibration points with the system-measured coordinates, the maximum error of the system’s three-dimensional spatial positioning was 0.08 m, which did not exceed the allowable error range.

#### 3.4.2. Radiation Field Simulation Verification

To verify the credibility of the system’s radiation field simulation, this study conducted a radiation field monitoring experiment in a nuclear-related laboratory using a Cs-137 radiation source. The source coordinates were (8, 6.5, 1), the *γ*-ray energy was 661.7 keV, and the activity was 1.24 × 10^6^ Bq. Dose rates were measured at four calibration points, and the measured values were compared with the system-simulated values. The data are presented in Table 3.

Considering that instrument measurements in radiation dose monitoring also introduce errors, the relative error in this study is defined as the relative error between the system-simulated values and the calibration true values. The mean relative error of the system-simulated values is 4.81%, and the maximum is 7.8%. Dose-rate monitoring is subject to multiple uncertainty factors related to the radiation source, monitoring equipment, and other aspects. According to GBZ 128-2002 [36], the allowable deviation between personal dose equivalent and environmental *γ* dose-rate monitoring results and the true values is ±20%. The deviations between the system-simulated values and the calibration true values do not exceed the allowable error range for personal and environmental radiation monitoring; therefore, such radiation field simulation errors are considered acceptable for radiation detection simulation equipment.

### 3.5. System Performance Comparison and Verification

Based on the Arduino UNO R3 as the core controller of the simulated detection device, the MPU6050 inertial sensor is used to acquire and process attitude and motion information, the HC-05 Bluetooth module enables wireless communication between the host controller and the simulated detection device, and the LCD12864 liquid crystal display updates and shows radiation dose-rate data in real time. The MPU6050 integrates a Kalman filtering algorithm and DMP function to output and fuse accelerometer and gyroscope data, thereby correcting and compensating for multidimensional data such as acceleration, angular velocity, and three-axis spatial rotation angles. According to the principle of transformation between the spatial inertial coordinate system and the target carrier coordinate system, the error-corrected angular-velocity data are mapped onto the inertial coordinate axes of the detection device during movement; through integration and a dynamic compensation mechanism, the system calculates the spatial displacement in the three axes to obtain the real-time spatial position of the detection device. Finally, combined with the radiation dose-rate calculation model, it receives dose-rate data from the host controller and displays them on the LCD screen with a refresh frequency of once per second. In three-dimensional spatial positioning, the mean absolute error does not exceed 0.06 m, the maximum error is controlled within 0.08 m, and the standard deviation of positioning error for repeated measurements at the same calibration point is less than 0.01 m to ensure stability and consistency of training data. In addition, the mean relative error between the simulated *γ* dose-equivalent rate and the standard true value is no more than 8%, and the maximum relative error is no more than 7.8%, which is better than the ±20% allowable error range specified in GBZ 128-2002. A comparative analysis between the proposed method and mainstream methods is shown in Table 4.

Compared with the other two methods, the relative improvement in detection efficiency regarding the maximum three-dimensional positioning error of this study is 46.7% and 86.3%, respectively, while the maximum relative error of radiation dose rate simulation is 7.8%. In summary, the system demonstrates clear advantages in safety and achieves compliance with the required accuracy, fully meeting the demands of closed-space nuclear radiation detection training.

## 4. Conclusions

In response to the practical needs of radiation source detection in nuclear accident emergency training, this paper proposes a design scheme and system prototype of penetrating radiation field simulation and detection equipment. Based on modeling the nuclear fuel through-radiation field, the system uses the inertial sensor MPU6050 (serial interface module) to acquire real-time three-axis acceleration and three-axis angular velocity. The main controller receives, processes, and analyzes the data to localize the simulated detection device within the preset radiation field and transmits the position information to the host computer. The host computer then performs integrated processing and sends the radiation intensity value to the Arduino controller to drive its screen display module and show the radiation intensity. The maximum three-dimensional positioning error is 0.08 m. Compared with other classical methods, the relative improvement in detection efficiency regarding the maximum three-dimensional positioning error of this study is 46.7% and 86.3%, respectively. The maximum relative error of the radiation dose rate simulation is 7.8%. The system’s radiation field simulation and radiation source localization accuracy can meet the requirements of nuclear emergency training. It achieves a balance between safety, economy, timeliness, and other aspects, providing a high cost-effectiveness technical means for nuclear emergency radiation detection training. Through trial use, some deficiencies have been identified in the current system. Future optimizations will be carried out in the following aspects to better support nuclear emergency radiation detection training:

(1) In the present study, the system only considers the simulated detection of the penetrating radiation field from a point source. Under accident conditions, the morphology and size of radiation sources may affect emergency response. Subsequent research should incorporate simulated training for radiation sources with different configurations and emitting rays of varying energies.
(2) Owing to the long computation time of the SuperMC program for radiation-field simulation, the preparation time for emergency training using the system is relatively long. Subsequent research should explore methods and measures to improve computational efficiency.
(3) Future studies will focus on testing dynamic scenarios involving variable velocity and rotation to verify the system’s real-time performance under dynamic conditions and to enhance the authenticity and effectiveness of training. At the same time, research will emphasize deep learning-based methods [39] for the reliability assessment of radiation fields.

## Figures and Tables

**Table 1:** Overall device parameters.

Device	Arduino Main Control Board Module	Device	MPU6050 Inertial Module
Processor	ATMega328P	IC	MPU-6050
1IO pin output current	20 mA	Communication	Standard IIC
Clock frequency	16 MHZ	Pin pitch	2.54 mm
Operating voltage	5 V	Supply voltage	3–5 V
External supply	9 V	Acceleration range	±16 g

**Table 2:** Statistical analysis of positioning error.

Point Serial Number	True Coordinate/m			System Measured Coordinate/m			System Measurement Error/m			
	*X*_0_	*Y*_0_	*Z*_0_	*X*_1_	*Y*_1_	*Z*_1_	ΔX	Δ*Y*	Δ*Z*	*D*
1	0.00	0.00	1.00	0.00	0.00	1.00	0.00	0.00	0.00	0.00
2	3.00	2.99	1.00	3.01	3.00	1.00	0.01	0.01	0.00	0.02
3	6.01	3.02	1.00	5.96	2.99	1.00	0.05	0.03	0.00	0.06
4	8.98	3.00	1.00	9.02	3.03	0.98	0.04	0.03	0.00	0.05
5	12.02	2.99	1.00	11.96	2.95	1.01	0.06	0.04	0.01	0.07
6	11.99	6.01	0.50	11.95	6.07	0.51	0.04	0.06	0.01	0.07
7	12.00	9.02	0.50	12.05	8.96	0.51	0.05	0.06	0.01	0.08
8	8.99	9.01	0.50	9.05	9.03	0.50	0.04	0.04	0.00	0.06
9	6.02	9.00	0.50	5.99	9.07	0.51	0.03	0.07	0.01	0.08
10	3.01	8.98	0.50	2.97	9.03	0.52	0.05	0.02	0.02	0.06

**Table 3:** Statistical analysis of dose rate error.

Point Serial Number	Coordinate of Marked Point/m			Absorbed Dose Rate/(μSv/h)			Relative Error
	*X*	*Y*	*Z*	Calibrated True Value	Measured Value	System Simulation Value	
1	4.00	6.50	1.00	0.0242	0.0283	0.251	3.7%
2	6.00	6.50	1.00	0.0968	0.0893	0.1034	6.8%
3	9.00	6.50	1.00	0.387	0.356	0.363	6.2%
4	11.00	6.50	1.00	0.0485	0.0564	0.0523	7.8%
5	8.00	3.50	1.00	0.0485	0.0547	0.0461	4.9%
6	8.00	6.00	1.00	1.548	1.446	1.508	2.6%
7	8.00	8.00	1.00	0.172	0.152	0.182	5.8%
8	8.00	10.50	1.00	0.0242	0.0272	0.253	4.5%
9	8.00	6.50	1.50	1.548	1.601	1.493	3.5%
10	8.00	6.50	2.00	0.387	0.402	0.396	2.3%

**Table 4:** Performance comparison of different radiation detection training devices.

Performance Indicators	This System	UWB System [37]	PDR-Based System [38]
Maximum error in three-dimensional positioning	Maximum 0.08 m	Maximum 0.15 m	Maximum 0.48 m
Anti-shielding capability in an enclosed space	Strong	Weak	Medium
Safety (without real radioactive sources)	High	High	High

## Data Availability

Data sharing does not apply to this article.
